# Stability toward High Energy Radiation of Non-Proteinogenic Amino Acids: Implications for the Origins of Life

**DOI:** 10.3390/life3030449

**Published:** 2013-07-30

**Authors:** Franco Cataldo, Susana Iglesias-Groth, Giancarlo Angelini, Yaser Hafez

**Affiliations:** 1 Actinium Chemical Research srl, Via Casilina 1626A, Rome 00133, Italy; 2 Astrophysical Observatory of Catania, Via S. Sofia 78, Catania 95123, Italy; 3 Astrophysical Institute of Canary Islands, Via Lactea s/n, La Laguna 38200, Tenerife, Spain; E-Mail: sigroth@iac.es; 4 Institute of Chemical Methodologies, CNR, Via Salaria Km 29,300, Monterotondo Stazione, Rome 00016, Italy; E-Mail: giancarlo.angelini@cnr.it; 5 National Center for Astronomy, KACST, P.O. Box 6086, Riyad 11442, Saudi Arabia; E-Mail: yafez@kacst.edu.sa

**Keywords:** amino acids, non-proteinogenic, radiolysis, degradation, stability, optical activity, racemization

## Abstract

A series of non-proteinogenic amino acids, most of them found quite commonly in the meteorites known as carbonaceous chondrites, were subjected to solid state radiolysis in vacuum to a total radiation dose of 3.2 MGy corresponding to 23% of the total dose expected to be taken by organic molecules buried in asteroids and meteorites since the beginning of the solar system 4.6 × 10^9^ years ago. The radiolyzed amino acids were studied by FT-IR spectroscopy, Differential Scanning Calorimetry (DSC) and by polarimety and Optical Rotatory Dispersion (ORD). It is shown that an important fraction of each amino acid is able to “survive” the massive dose of radiation, while the enantiomeric excess is partially preserved. Based on the results obtained, it is concluded that it is unsurprising to find amino acids even in enantiomeric excess in carbonaceous chondrites.

## 1. Introduction

One of the most fascinating results of astrochemistry and astrobiology was the discovery that certain meteorites and in particular the carbonaceous chondrites are incredibly rich with molecules belonging to different classes of organic compounds and that most of them can be considered the precursors or key “building blocks” of the more complex molecules used for life [1,2,3,4,5,6,7,8,9,10,11,12,13,14,15,16,17]. Among the various classes of molecules found in carbonaceous chondrites, the amino acids found there are extremely important because some of them were found as scalemic mixtures, prevalently with the L enantiomer in slight excess [8]. Another characteristic feature of the amino acids found in meteorites regards the fact that they are in large part not used by terrestrial biochemistry, thus they are non-proteinogenic amino acids completely extraneous to the biosphere [3,4,5,6,7]. More precisely, in very few years, the identified amino acids in carbonaceous chondrites passed from 66 [7] to more than 100 different molecular species [11,14]. However, only a minor fraction of these amino acids, approximately 10%, are currently used by the terrestrial biochemistry [7,11,14]; all the others are not encoded in DNA and hence are not involved in protein synthesis [7]. From one point of view, this fact is extremely important because it excludes categorically the contamination of the carbonaceous chondrites analyzed by terrestrial life; otherwise, one would have found only the 21 terrestrial and proteinaceous amino acids. There are other aspects which support the non-terrestrial origins of the amino acids found in meteorites, such as the high D and ^13^C isotopic enrichment [9,15], or even the distribution of amino acid products which are completely compatible with an abiotic synthesis starting from simple molecules like methane, ammonia, carbon dioxide and UV irradiation or corpuscular radiation bombardment [6,18,19,20]. Indeed, experiments using circularly polarized light, for instance, from a synchrotron is able not only to produce a mixture of amino acids but also in scalemic mixtures [6,20]. Thus, a typical scenario for the synthesis of the amino acids involves the UV irradiation of presolar nebula in space with formation of complex molecules including amino acids [14]. If the UV light source in space is not circularly polarized, the amino acids are formed in racemic mixture [21,22]. However, irradiation or racemic mixtures of amino acids with circularly polarized light, for instance from certain neutron star sources [6,21,22,23], leads to the formation of a scalemic mixture by the selective and preferential destruction of one enantiomer against the other which accumulates creating a scalemic mixture [21,22,23]. Once formed in the organic clouds of the interstellar medium, the amino acids and other organic molecules are incorporated in asteroids, comets and other bodies during the formation of the solar system which then deliver their precious load to the earth through the meteoritic and cometary bombardment age [14,21,24,25]. Certain bodies in the external part of the solar system in the Oort cloud, in the Kuiper belt and beyond could be very primitive without important alterations since the time of their aggregation during the formation of the solar system 4.6 × 10^9^ years ago. Consequently, also the organic molecules present in these bodies were not subjected to important alterations other than cosmic ray radiation and radionuclide decay radiation. Water alteration may occur as discussed widely by various authors [9,10,13]. However, in our study, we will not consider the effect of water. The problem of radiation processing of organic molecules embedded in asteroids and comets was addressed for the first time by the Nobel laureate Harold Urey more than 60 years ago [26,27,28], but the result of his calculation is still valid today [29,30]. Urey stated that organic molecules buried at a depth of 20 m are completely shielded by cosmic ray radiation and can be preserved unchanged for billions years. Under these conditions, the main decomposition source for these molecules is due to the ^26^A1, ^40^K, ^235^U, ^238^U and ^232^Th radionuclide decay which release a total radiation dose of about 14 MGy in 4.6 × 10^9^ years [27,28,29]. The delivery of such energy is not linear but is very high in the first billion of years of the solar system due to the decay of the radionuclide ^26^A1 which is characterized by a short half-life in comparison to the other aforementioned radionuclides characterized by much longer half-lives [31]. In order to assess the radiolysis resistance of amino acids and their preservation of chirality to high energy radiation, we have started a systematic study on the radiolysis of all proteinogenic amino acids to a radiation dose of 3.2 MGy, which corresponds to 22.8% of the total dose they should have taken inside asteroids, comets and other bodies of the solar system [30,32,33,34,35]. The results were extrapolated to 14 MGy of radiation dose, showing that practically all the proteinogenic amino acids could partially “survive” such an enormous radiation dose with preservation of the chiral excess [35]. Based on our study, it is therefore no surprise to have found amino acids in carbonaceous chondrites and also in enantiomeric excess. As discussed previously, only 10% of the total amino acid species found in meteorites are proteinogenic [7,11,14]. We thus decided to irradiate in the solid state to 3.2 MGy a series of unusual amino acids belonging to the classes found in carbonaceous chondrites; the preliminary results were published elsewhere [36], while the present work represents a full account of our results. The present work is part of our long-term research project on chirality in terms of chiral polymer synthesis, chiral induction, and amplification [37,38,39,40,41,42,43,44].

## 2. Experimental Section

### 2.1. Materials and Equipment

**Scheme 1:**
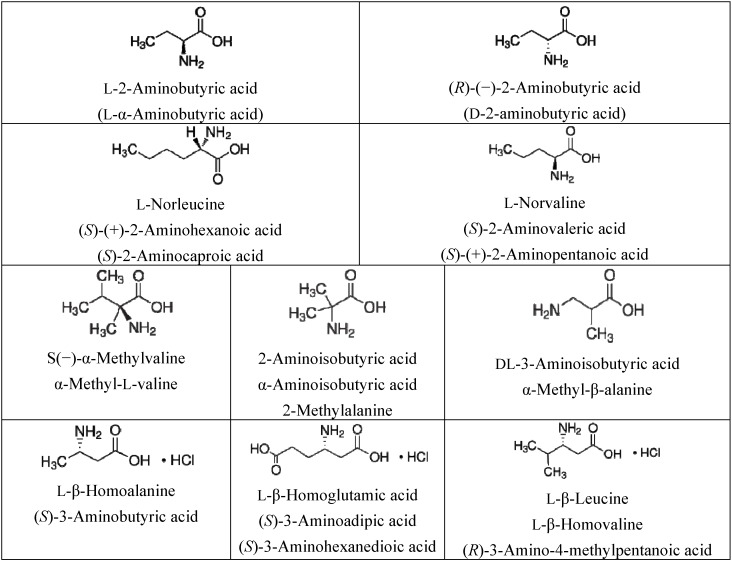
Chemical structures of the amino acids studied in the present work.

The amino acids l-2-aminobutyric acid, d-2-aminobutyric acid, 2-aminoisobutyric acid (or α-aminoisobutyric acid), l-norleucine, l-norvaline, l-β-leucine HCl, l-β-homoalanine HCl, l-β-homoglutamic acid HCl, S(−)-α-methylvaline and dl-3-aminoisobutyric acid (all shown in Scheme 1) were obtained from Sigma-Aldrich (Milan, Italia) and used as received. The differential scanning calorimetric analysis (DSC) of the amino acids before and after irradiation was made on a DSC-1 Star System from Mettler-Toledo. The Optical Rotatory Dispersion (ORD) spectra were obtained on a Jasco P-2000 spectropolarimeter with a dedicated monochromator. The FT-IR spectra were recorded on a Thermo-Scientific infrared spectrometer model Nicolet-6700 in reflectance mode using a ZnSe crystal as spectral window and sample support.

### 2.2. Irradiation Procedure with γ Rays

The irradiation with γ rays was made in a ^60^Co Gammacell 220 from Atomic Energy of Canada at a dose rate of 1.5 kGy/h. A total dose of 3,200 kGy = 3.2 MGy was delivered to each sample. Each weighed amino acid of this study was transferred, prior to irradiation, into a Durhan borosilicate glass vial which was connected to a high vacuum line. Each flask was sealed under high vacuum and irradiated at a dose rate of 1.5 kGy/h. Thus, all irradiations were conducted under high vacuum. After the radiolysis, the flasks were opened and the content weighed again in order to determine the amount volatilized due to radiolysis. At this stage weighing was not very accurate because the irradiated amino acids trap free radicals and tend to be electrified and to stick on the glass walls.

### 2.3. Analysis with Differential Scanning Calorimetry

The irradiated samples were tested for purity by a DSC at a heating rate of 10 °C/min under N_2_ flow. As reference the DSC test was applied also on pristine, non-irradiated samples under the same conditions. The amount % of residual sample after the solid state radiolysis Nγ was determined from the ratio of the melting enthalpy after the radiolysis at 3.2 MGy (ΔH_γ_) and the enthalpy before radiolysis measured on the pristine sample (ΔH_0_):

Nγ% = 100 [ΔH_γ_/ΔH_0_] (1)

The DSC trace on all amino acids under study was recorded on conventional Al crucibles with a pierced cap. However, for some amino acids (reported with an asterisk in Table 1) the DSC scan was also repeated in a stainless steel sealed crucible to avoid premature decomposition, an approach already applied for aromatic amino acids [32]. Also in this case, Nγ% was determined according to Equation (1).

**Table 1:** Summary of results of the amino acids studied in the present work.

	Equation 3	Equation 2	Pristine	3.2 MGy	Equation 1	σ		
Amino Acid	Rα	Rγ	ΔH_0_ melt. (J/g)	ΔH_γ_ melt. (J/g)	Nγ%	Standard deviation 3 × 3 measu-rement	Residual weight after radiolysis (%)	Notes after radiolysis
(*)2-Aminoisobutyric acid [α-AIB]	n.a.	n.a.	1081.0	944.8	87.4	1.1	97.6	yellow, aminic and fatty odor
(*) dl-3-aminoisobutyric a. [dl-3-AIB]	n.a.	n.a.	180.3	153.5	85.1	1.5	95.4	pale yellow aminic odor
(*) d-2-aminobutyric acid [d-ABA]	78.6	75.0	796.3	601.0	75.5	0.5	97.2	dark yellow, aminic and fatty odor
(*) l-2-aminobutyric acid [l-ABA]	64.3	73.7	752.1	543.1	72.2	4.6	92.8	yellow, aminic and fatty odor
(*) l-β-homoalanine HCl [β-Ala]	84.2	86.4	476.0	365.0	76.7	n.d.	95.9	brown, sweet odor; DSC shows melting with decomposition
l-β-homoglutamic acid HCl [β-Glu]	91.3	93.4	180.2	134.8	74.8	n.d.	94.0	cream color; DSC only melting peak considered
(*) l-norleucine [Nle]	63.6	70.8	681.4	488.5	71.7	n.d.	93.0	light yellow, weak aminic odor
(*) l-norvaline [Nvl]	81.0	84.0	782.1	549.0	70.2	n.d.	96.8	pale yellow, distinctive aminic odor
S(−)-α-Methylvaline [α-MV]	88.7	92.8	850.9	595.2	69.9	n.d.	95.8	pale yellow, pungent odor
l-β-leucine HCl [β-Leu]	74.3	79.9	138.9	74.2	53.4	n.d.	94.5	cream color, sweet odor; DSC only melting peak considered

(*) measured in steel sealed crucible.

### 2.4. Analysis of the Radioracemization Degree by Optical Rotatory Dispersion Spectroscopy

The degree of radioracemization (intended as the residual optical rotation after radiolysis) was measured by ORD spectroscopy [45]. The irradiated amino acid samples were dissolved in HCl 1 M (about 80 mg/10 mL) and the optical rotation was measured on the resulting solution using a polarimetric cell of 0.5 dm length in the range between 375 and 600 nm. As reference, the same measurement was also made on standard pristine amino acid dissolved in the same medium at the same concentration. From the ratio of the average specific optical rotation after radiolysis [a]γ and before radiolysis [a]_0_ the residual optical activity Rγ was determined [32,33,34,35,36]:

Rγ% = 100{[α]_γ_/[α]_0_} (2)

Furthermore, the radioracemization at the D line of sodium (at 589 nm) was determined using an equation similar to Equation (2), but using the specific optical rotation after radiolysis [α]_Dγ_ and before radiolysis [α]_D0_, so that:

Rα% = 100{[α]_Dγ_/[α]_D0_} (3)

### 2.5. FT-IR Analysis of the Amino Acids

The infrared spectra of all the amino acids samples studied in the present work were recorded before and after the radiolysis at 3.2 MGy. The spectra recorded were normalized with the Omnic software of the spectrometer and subtracted each other obtaining the difference spectra putting in evidence the major changes occurred.

## 3. Results and Discussion

### 3.1. Amino Acids Selection

After having studied the radiolysis stability of all proteinogenic amino acids in an astrochemical perspective [30,32,33,34,35], we are focusing our attention to non-proteinogenic or non-proteinaceous amino acids which are abundant in carbonaceous chondrites exceeding by far the concentration and the variety of proteinogenic amino acids [1,2,3,4,5,6,7,8,9,10,11,12,13,14,15,16,17]. Here we report briefly the list of the selected amino acids for the present work explaining the reason for their selection. It must be underlined here that we based our selection essentially on the basis of the amino acids found in the CM2 chondrites Murchison and Murray reviewed in ref. [3,7,46]. However, recent work by Glavin and Dworkin and co-workers at NASA [13,14,15,17] and Martins and colleagues [10,16] has shown that the amino acid abundance in CM2 chondrites is not representative of all carbonaceous chondrites.

α-Aminoisobutyric acid (α-AIB), which is known also as 2-aminoisobutyric or 2-methylalanine (see Scheme 1), was selected because it is the most abundant amino acid found in Murchison (representing up to 20% of the total amino acids amounts) and in other meteorites exceeding often also glycine, which is the other most common and abundant amino acid in carbonaceous chondrites [2,7,46]. α-AIB is not a chiral amino acid and is not DNA-encoded for protein synthesis although it has strong helix-induction properties once incorporated in polypeptides [47]. Another α-methyl amino acid studied in the present work is S(−)-α-methylvaline (α-MV) (see Scheme 1), which is chiral and which ranks 14th in terms of abundance in the Murchison meteorite [46]. As shown in Scheme 1, α-methyl amino acids like α-AIB and α-methylvaline are structurally different from common α-H amino acids of terrestrial biology due to the α-carbon atom bearing the carboxylic and amino functionality. It also bears a methyl radical rather than a hydrogen atom. This fact makes the α-methyl amino acids much less prone to racemization and to hydrolytic degradation in aqueous solution than the corresponding α-H amino acids [7]. 

l-2-aminobutyric acid (l-α-ABA) and d-2-aminobutyric acid (d-α-ABA) together with l-norleucine (l-Nle) and l-norvaline (l-Nvl) (see Scheme 1) have the chemical structures of alkyl-type α-H-amino acids, analogous to that of the proteinogenic amino acids. All the aforementioned α-H-amino acids, although biologically active, are not common in the terrestrial biochemistry and are not encoded in DNA for protein synthesis [47].

However, these amino acids were found in Murchison meteorite, and α-ABA ranks 7th in abundance among the amino acids found in that carbonaceous chondrite [46]. An enantiomeric excess of l-α-ABA of 4% was detected in Murchison meteorite [2,7] and both l-α-ABA and d-α-ABA were detected in the amino acid mixture produced with circularly polarized light irradiation of simulated interstellar ices [6].

β-Amino acids are another class of biologically active molecules where the amino functionality is attached to a carbon atom in β position with respect to the carboxylic functionality. This is in contrast to α-amino acids where both the amino and carboxylic functionalities share the same carbon atom. In this study, we have selected three chiral β-amino acids which were irradiated in the solid state as HCl salts. The selected β-amino acids are: l-β-homoalanine (l-β-Ala), l-β-homoglutamic acid (l-β-Glu)and l-β-leucine (l-β-Leu)(see Scheme 1). Another β-amino acid used in the present study is dl-3-aminoisobutyric acid (dl-3-AIB) in its racemic form. The reason for selecting β-amino acids resides in the fact that they were found in Murchison and Murray meteorites in significant l-enantiomeric excess [3].

### 3.2. Non-Proteinaceous Amino Acids Radiolysis: Infrared Difference Spectroscopy

The radiolysis of amino acids was studied in the past for various reasons ranging from food sterilization [48,49] to radiation dose determination [50], taking advantage of the fact that amino acids are able to form stable radicals [51,52,53,54,55]. About amino acids radiolysis, there are available also the very important and classical works of Bonner and colleagues [21,56,57,58,59,60], which were specifically designed to test the Wester-Ulbricht hypothesis that parity violation in the β-decay may have originated the enantiomeric excess in the biochemical world, and the work of Kminek and Bada [61]. However, with the exclusion of Cataldo and colleagues [30,32,33,34,35,36], no systematic studies on the effects of high energy radiation on amino acids have been made. This paper represents a continuation of the work.

**Figure 1:**
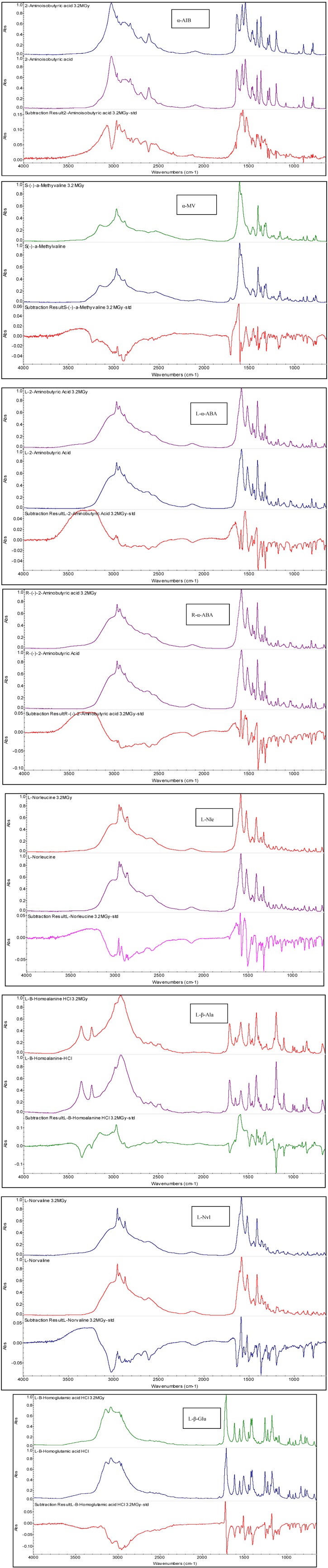
FT-IR spectra of amino acids studied in the present work. Each panel shows first the spectrum taken after radiolysis at 3.2 MGy, then the spectrum of the pristine amino acid and, finally, at the bottom of the panel, the difference between the two spectra. The peaks pointing upward are due to the radiolyzed sample, those pointing downward are due to the reference sample.

**Figure 2:**
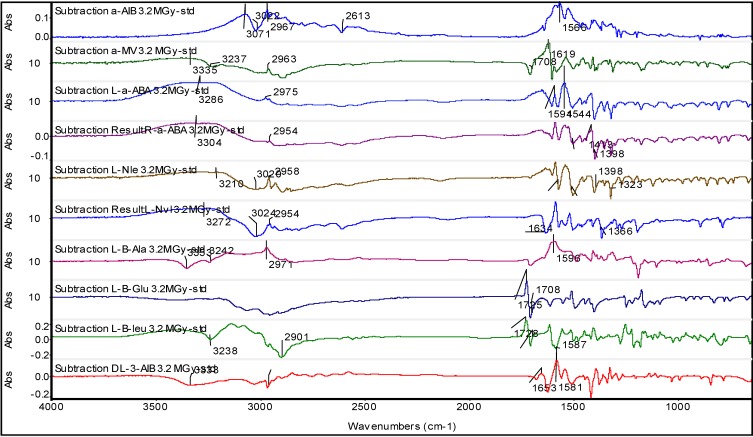
Difference FT-IR spectra obtained by subtracting the reference amino acid infrared spectrum to that of the radiolyzed amino acid at 3.2 MGy. From top to bottom are reported the difference spectra of α-AIB, α-MV, l-ABA, d-ABA, l-Nvl, l-Nle, l-β-Ala, l-β-Glu, l-β-Leu and dl-3-AIB.

The FT-IR spectra of the irradiated amino acids selected for this study are shown in Figure 1. Each panel shown in Figure 1 is referring to a specific amino acid (from top to bottom and from left column first to the right column last) namely: α-AIB, l-α-ABA, l-Nle, l-Nvl, R-α-ABA, l-β-Ala and l-β-Glu; the spectra of l-B-Leu and dl-3-AIB are not shown in Figure 1 but are completely analogous to those shown. Inside each panel of Figure 1 are reported from top to bottom the infrared spectrum taken after radiolysis at 3.2 MGy in vacuum in comparison to the pristine reference amino acid and the difference of the two spectra. It is evident at first glance that despite the massive radiation dose administered to the amino acids the spectral changes are subtle and can be detected only through the difference of the two spectra: radiolyzed minus reference. The detailed difference spectra are reported in Figure 2.

It is necessary to use the infrared difference spectroscopy [62] to put in evidence the relatively small spectral changes occurred after the massive radiolysis at 3.2 MGy in the solid state under vacuum. These changes are evident in Figure 2 for each amino acid studied. In the difference spectra, the bands pointing upward belong to the radiolyzed samples and indicate a growth of these bands after radiolysis. Conversely, the bands pointing downward are due to the reference amino acid and were reduced in intensity or disappeared after the radiolysis. From the spectra of Figure 2 it is evident that the amino acid functionalities more affected by the radiolysis is the amino group which in the solid state amino acid is always in protonated form *i.e.*, -NH_3_^+^ because the amino acids in the solid state are exclusively in zwitterionic form [63,64,65], which for α-H amino acids can be represented as ^+^NH_3_-CH(R)-COO^-^. The -NH_3_^+^ gives a broad multiplet in the amino acids spectra in the 3,300–2,600 cm^−1^ range [63]. To clarify, the asymmetric stretching occurs as a broad band at 3,130–3,030 cm^−1^ but can be shifted at higher frequency if in the form of HCl salt [63,64]. Instead, the symmetric stretching of -NH_3_^+^ group occurs in the broad region comprised between 3,000 and 2,000 cm^−1^ together with combination bands and is generally weaker than the asymmetric stretching and of less diagnostic value [63,64] Indeed, all the difference spectra shown in Figure 2 show profound changes just in the spectral region of the asymmetric -NH_3_^+^ stretching, confirming that the radiolysis was affective principally in this group. Furthermore, the bending vibration mode of -NH_3_^+^ which can be distinguished in asymmetric and symmetric occurs in the range comprised between 1,665–1,585 cm^−1^ and 1,550–1,470 cm^−1^, respectively [63,64]. Once again, the difference spectra of Figure 2 show clear evidences of changes in the absorption bands just in the -NH_3_^+^ bending region. All these results are consistent with the delicate and elegant studies made in the 1970s [48,49] which have shown that the predominant reaction in the solid state radiolysis of practically all the aliphatic amino acids is the release of ammonia with radiation chemical yield G(NH_3_) ≈ 5 molecules/100 eV. The release of ammonia leads necessarily to the formation of carboxylic acids with G ≈ 2.5 molecules/100 eV and and keto-acids 2.5 ≥ G ≥ 1.1 (in molecules/100 eV). The reactions involved, following Garrison [49] are:

^+^NH_3_-CH(R)-COO^−^ → ^+^NH_3_-C^•^(R)-COO^−^ + H^+^ + e^−^ (4)

2 ^+^NH_3_-C^•^(R)-COO^−^ → ^+^NH_2_=C(R)-COO^−^ + NH_2_-CH(R)-COO^−^ (5)

^+^NH_2_=C(R)-COO^−^ + H_2_O → NH_4_^+^ + R-CO-COO^−^ (6)

^+^NH_3_-CH(R)-COO^−^ + e^−^ → ^+^NH_3_-CH(R)-C^•^OO^2−^ → NH_3_ + ^•^CH(R)-COO^−^ (7)

^•^CH(R)-COO^−^ + ^+^NH_3_-CH(R)-COO^−^ → R-CH_2_-COO^−^ + ^+^NH_3_-C^•^(R)-COO^−^ (8)

The formation of CO_2_ in the solid state radiolysis of amino acids is by far a secondary reaction in comparison to the production of ammonia. In fact, the radiation chemical yield for the CO_2_ production is only G(CO_2_) ≈ 1 molecules/100 eV and approximately unitary radiation chemical yields are reported for the amine and amide production while the release of H_2_ and mixture of hydrocarbons is quite negligible with 0.5 ≥ G ≥ 0.1 (in molecules/100 eV) [48,49]. From the difference spectra of Figure 2 it is difficult to have a clear evidence of the secondary decaboxylation reaction of amino acids also because the asymmetric -COO^−^ stretching occurs at 1,605–1,555 cm^−1^ with a significant overlap to the -NH_3_^+^ asymmetric bending region while the symmetric -COO^−^ stretching occurs in the 1,425–1,390 cm^−1^ region and it is much weaker than the asymmetric stretching [34,63].

It is expected that also the radiolysis of α-methyl amino acids and β-amino acids should yield ammonia as the main radiolysis product [54].

### 3.3. Amino Acids Weight Loss after 3.2 MGy Radiolysis

As discussed in the previous section, the amino acid radiolysis causes the release of NH_3_, carboxylic and keto acids, and other minor products like CO_2_, amines/amides, mixtures of hydrocarbons where, in general, methane is predominant in addition to hydrogen. Being conducted in the solid crystalline state under vacuum, the radiolysis products escape from the crystalline structure of the amino acids at a different speed, depending essentially on the molecular weight of the radiolysis products. Thus, it is obvious to think that H_2_, CH_4_, NH_3_ are easily released from the crystalline cage while radiolysis products having higher molecular weight are released more slowly because it is more reasonable that they remain trapped in the crystalline cage. Indeed, as annotated in Table 1, all the radiolyzed amino acids release an amine and fatty smell which is connected with the production and release of ammonia and fatty and keto-acids derivatives.

The weight of each amino acid was measured before radiolysis and, although not very accurate (see Section 2.2.), the residual weight was measured again after radiolysis at 3.2 MGy. In all cases, a weight loss was detected as expected and the residual weight of each amino acid is reported in Table 1 as % of the starting weight. Thus, the weight loss of α-AIB was 2.4% and the residual weight after radiolysis 97.6% of the starting weight before radiolysis. The weight loss is relatively small for α-AIB which indeed shows also discrete residual purity of 87.4% after radiolysis as measured by DSC according to Equation (1). Instead, the radiolysis weight loss appears more pronounced in the case of l-Nle and l-β-Leu reaching 7% of the original starting weight and also for l-ABA, which will be discussed in the next section.

It is curious to note that α-AIB which appears to be the most stable amino acid toward radiolysis, is also the most abundant amino acid found in carbonaceous chondrites.

### 3.4. Residual Purity Measurement of Amino Acids after 3.2 MGy Radiolysis

The purity of organic chemicals can be determined through the melting enthalpy of a given compound using the differential scanning calorimetry (DSC) [66,67]. The practical approach for the determination of the purity of a given compound makes use of Equation (1) where the purity is determined by the ratio of the melting enthalpies measured in the same conditions for the impure compound (which contains the radiolysis products) against the melting enthalpy of the 99% pure reference compound. Thus, for each amino acid used in the present study, the melting enthalpy was determined first for the reference pure compound (ΔH_0_) and then re-determined on the same compound after the solid state radiolysis at 3.2 MGy in vacuum (ΔH_γ_). The results of such determination are reported in Table 1. By substituting these results in Equation (1), the residual amount of a given amino acid after radiolysis Nγ% was determined and reported again in Table 1 where all the amino acids studied are ordered from the most radiostable to the less stable toward high energy radiation.

In the previous preliminary work [36] the purity of the radiolyzed amino acids was determined through Equation (1), but the measurements were made in conventional aluminum crucibles with punched cap. However, since most of the amino acids studied melt above 300 °C (see Table 2), the measurement of the melting enthalpy was affected by a parallel thermo-oxidative degradation of a given amino acid. Consequently, in the present study, all the DSC measurements were repeated for all amino acids using stainless steel sealed crucibles with the exception of β-Glu and β-Leu which are characterized by a relatively low melting points.

**Table 2:** DSC melting points before and after radiolysis.

Amino Acid	Abbreviation	Melting point °C pristine reference	Melting point °C after radiolysis	Melting point shift after radiolysis
2-Aminoisobutyric acid (*)	α-AIB	340.3	327.7	−12.0
dl-3-aminoisobutyric a. (*)	dl-3-AIB	172.1	171.2	−0.9
d-2-aminobutyric acid (*)	d-ABA	301.3	290.0	−11.3
l-2-aminobutyric acid (*)	l-ABA	302.9	290.2	−12.7
l-β-Homolanine HCl (*)	l-β-Ala	244.5	243.5	−1.0
l-β-homoglutamic acid HCl	l-β-Glu	190.8	182.7	−8.1
l-norleucine (*)	l-Nle	322.9	297.4	−25.5
l-norvaline (*)	l-Nvl	321.2	302.6	−18.6
S(−)-α-Methylvaline	α-MV	306.1	298.8	−7.3
l-β-leucine HCl	l-β-Leu	176.3	159.8	−16.5

(*) measured in steel sealed crucible.

Furthermore, in order to assess the variability of the DSC measurement for the following amino acids: α-AIB, dl-3-AIB, d-ABA and l-ABA the DSC measurements were repeated three times on the reference pristine samples and three times on the radiolyzed samples. The standard deviation (σ) values of the resulting Nγ% are reported in Table 1. As expected, the σ values found for the former three amino acids are very small, implying (using {[±2σ/Nγ%]100}) that the Nγ% values reported in Table 1 are affected by ±3.5% uncertainty in the worse case, and only ±1.3% in the best case. Surprisingly, only for l-ABA a very high σ = 4.6 was found and it is not easily explainable unless advocating the effects of impurities present in it which gives this high and unusual variability.

**Figure 3:**
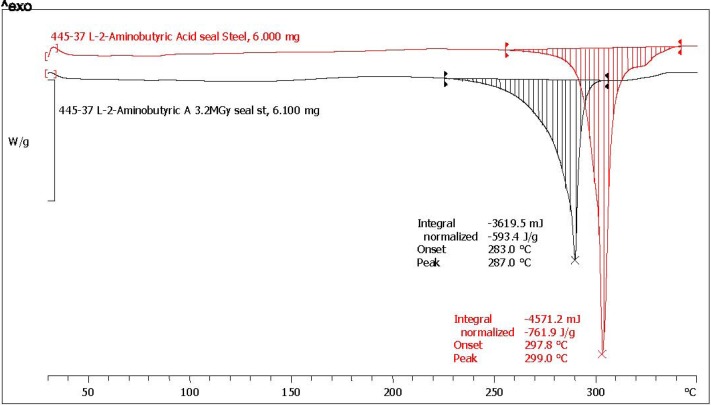
DSC trace of l-ABA in sealed stainless steel crucible at a heating rate of 10 °C/min. The red trace is due to pristine reference l-ABA while the black trace is due to l-ABA radiolyzed at 3.2 MGy.

**Figure 4:**
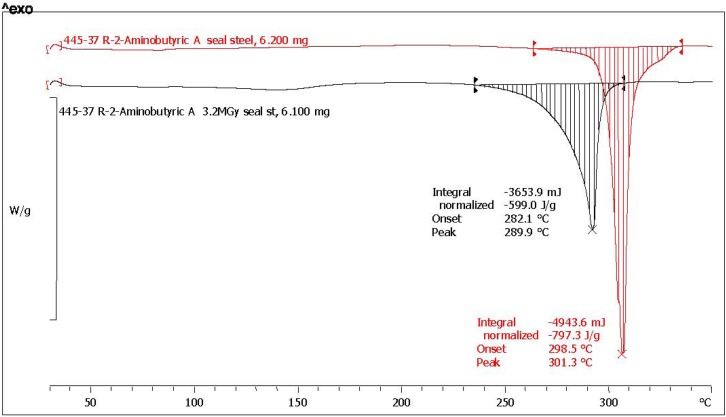
DSC trace of d-ABA in sealed stainless steel crucible at a heating rate of 10 °C/min. The red trace is due to pristine reference d-ABA while the black trace is due to d-ABA radiolyzed at 3.2 MGy.

The melting enthalpy is the amount of energy necessary to break down the crystalline structure of a molecular solid and hence is the amount of heat that must be supplied to the said molecular solid to cause the phase transition from solid to liquid. As already discussed in the previous section, the solid state radiolysis of amino acids causes a series of defects in the original crystalline structure. The defects can be explained as a consequence of the alteration of the molecular structure of the amino acids which release gaseous products like NH_3_, H_2_, CH_4_, CO_2_ but also because inside the crystalline structure are formed new products like carboxylic and keto acids, amines and amides which of course alter the crystal structure and act as impurities during the heating and melting process. The macroscopic consequences of the presence of the radiolysis products and of the presence of defects in the radiolyzed amino acids crystals are easily visualized at the DSC analysis as a broadening of the melting transition and as a reduction of the melting point onset and peak. These effects can be observed in Figure 3, Figure 4 where the DSC traces of l-ABA and d-ABA are reported, respectively. The melting transitions of pristine l-ABA and d-ABA are characterized by a sharp melting peak located respectively at 299 °C and 301 °C and the relative endothermal transitions are ΔH_0_ = 761.9 J/g and ΔH_0_ = 797.3 J/g respectively. Note that there is a small difference in the melting enthalpy of the two enantiomers studied, but this difference should be attributed more to the presence of small impurities in the two samples since the two enantiomers should display both the same melting point and the same melting enthalpy. Thus, based on these data, d-ABA appears more pure than l-ABA. Indeed the purity of d-ABA is >99% as determined by GLC as claimed by the supplier while l-ABA is >99%, though such purity was determined by titration which is not a sensitive analytical technique as GLC. Consequently, we have evidence also from the supplier that d-ABA was purer that l-ABA.

However, the melting enthalpies of pristine (and radiolyzed) l-ABA and d-ABA reported in Table 1 are not the same as those reported in Figure 3, Figure 4 because the latter represent just a single measurement while in Table 1 the average of three measurements each are reported. As already anticipated, also the averaged melting enthalpy of l-ABA results are lower than that of d-ABA as already suggested by the single measurements of Figure 3, Figure 4. Furthermore, the melting enthalpy data of d-ABA are very close each other, while in the case of l-ABA, there is a tendency for higher scattering as demonstrated by the σ values reported in Table 1.

In regards to Figure 3, Figure 4, the radiolysis of l-ABA and d-ABA has caused a significant alteration of the melting transition which in fact appears in both cases broadened in comparison to the melting transition of the pristine reference l-ABA and d-ABA. Furthermore, it is evident also a shift to lower temperatures of the melting point peak in both cases after radiolysis (see also Table 2 for temperature shift after radiolysis). The broadening of the melting transition after radiolysis is a consequence of the impurities formed from the radiation-induced decomposition of the radiolyzed amino acids and is detected as a reduction of the melting enthalpy which is now (see Table 1 the averaged values) ΔH_γ_ = 543.4 J/g for l-ABA and ΔH_γ_ = 601.0 J/g for d-ABA. Therefore, the residual purity of l-ABA after radiolysis is N_γ_% = (543.4/752.1)100 = 72.2% of the starting value, while for d-ABA, N_γ_% = (601.0/796.3)100 = 75.5% of the starting value. Such a procedure was adopted for all the amino acids studied and the results are reported in Table 1. It is interesting to note that d-ABA, which is purer than l-ABA, appears also more radiostable than its enantiomer and the N_γ_% values show a much lower standard deviation than in the case of l-ABA. It is important to point out here that the radiolysis of two enantiomers must always yield the same amount of residual, not radiolyzed, product. In other words, the radiolysis cannot be asymmetric, destroying preferentially one enantiomer over the other, unless special experimental conditions are adopted to try to get advantage of the parity violation in the β-decay to produce an asymmetric radiolysis as it was done by Bonner and colleagues without any positive results [21,56,57,58,59,60]. Since the radiolysis in our case was not conducted under any special irradiation conditions, since the asymmetric radiolysis due to parity violation in the β-decay has not been proved in a firm and reproducible way, the small differences in the residual amount of l-ABA and d-ABA we detect after 3.2 MGy are certainly due to the effects of impurities which, for example, give a slightly worse radiation stability to l-ABA accompanied by a high σ values in comparison to the d-ABA. In other words, the impurities present in l-ABA before the radiolysis facilitate the radiolytic effect causing also a remarkable scattering of the melting enthalpy values. On the contrary, d-ABA, which was purer than l-ABA before radiolysis, gives both before and after radiolysis an extremely narrow distribution of the melting enthalpy values and this is reflected both in the higher N_γ_% and in the extremely low σ = 0.5 value. These facts show how delicate the field of asymmetric radiolysis is and the incredible role that the impurities could have played in the preservation of a prebiotic amino acids exerting a radioprotection effect or facilitating the radiolysis and enhancing the radiolytic effects. We must reflect also about how far our laboratory conditions are from reality if we consider that millions of different organic compounds were detected in the Murchison meteorite and which may necessarily act as impurities of any biologically important compound [12]. Another consideration to be taken here regards the fact that from the radiolysis of a simple compound like a given amino acid, there is the formation of about 10 different classes of compounds and more than 20 different molecular species: what will happen when a complex mixture of 10^3^–10^4^ different compounds present in the asteroids at the beginning of the solar system 4.6 × 10^9^ years ago are radiolyzed? They must produce an incredible plethora of products reaching the incredible complexity observed in the meteorites where millions of different molecular species are discussed [12]. 

Figure 3, Figure 4 show also the melting point shift after radiolysis. l-ABA and d-ABA in pristine form melt respectively at 303 °C and 301 °C and after radiolysis at 3.2 MGy the melting points were found in both cases at 290 °C as summarized in Table 2 for all amino acids examined. The radiolysis products trapped in the amino acid crystals affect the melting point causing its reduction with a shift to lower temperatures of more than 11 °C. This is a general trend observed for all amino acid studied which show larger or smaller shifts but always toward lower temperatures after radiolysis as evidenced in Table 2.

**Figure 5:**
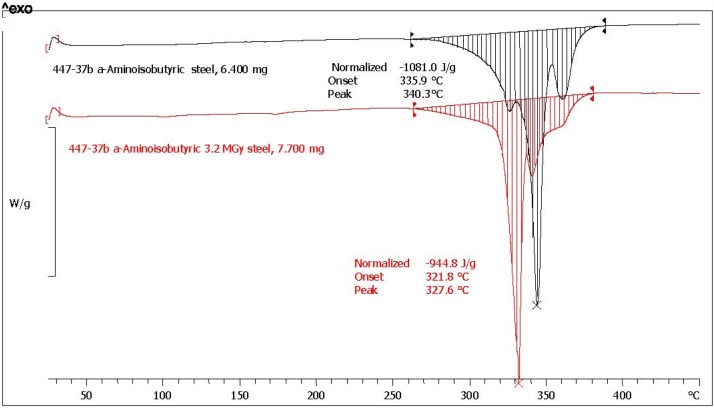
DSC traces of α-AIB recorded in sealed stainless steel crucible at a heating rate of 10 °C/min. In red is reported the trace of pristine reference α-AIB while in black it is reported the trace of α-AIB radiolyzed at 3.2 MGy.

As shown in Table 1, the amino acid showing the best radiation stability is α-AIB with N_γ_% = 87.4 and a low standard deviation σ = 1.1. Figure 5 shows the melting transitions of both a pristine and s radiolyzed sample. Both the melting transitions are sharp and, of course the melting transition of pristine α-AIB appears sharper and at higher temperature (340 °C) against the melting transition of the radiolyzed α-AIB whose melting point peak is at about 328 °C. From three measurements of melting enthalpy the average residual purity of the radiolyzed α-AIB has been re-calculated and reported in Table 1. In the previous work [36], the DSC traces of α-AIB pristine and radiolyzed were characterized by an extremely broad transition which has affected both the melting point peak position and the melting enthalpy leading to unreliable conclusions which are superseded by the present work. 

Looking at the radiolytic stability of all amino acids examined and reported in Table 1, no specific amino acid class with a special stability over the others seems to emerge. However, looking at the two α-methyl amino acids α-AIB and α-MV, it is necessary to observe the lack of hydrogen atom in the α-carbon atom of α-AIB and its presence in α-MV structure. This permits to the reaction of Equation (4) to occur easily in the case of α-MV together with all other reactions, Equations (5)–(8), causing its decomposition. In contrast, the radiolysis of α-AIB cannot follow the reaction of Equation (4) because of the lack of H atom in the α-carbon and hence the radiolytic decomposition occurs essentially mainly through to Equation (7). This explains why α-AIB has the best radiolyitic stability among the amino acids studied and perhaps this explains also why it is so abundant in the Murchison and Murray meteorites.

The radiolytic stability of α-H amino acids examined in this work is quite similar. The Nγ% value is slightly higher for d-ABA, l-ABA followed by Nle and Nvl, which are very close each other. In this case, the difference among these four amino acid is essentially the length of the aliphatic side chain. A general rule predicts lower radiolytic stability for amino acids with longer side chain as indeed observed since Nle and Nvl provided by a longer aliphatic side chain than d-ABA, l-ABA display a slightly lower radiolytic stability than the latter two.

Concerning β-amino acids of this study (dl-3-AIB, β-Ala, β-Glu and β-Leu), the amino functionality is attached to β-carbon atom of the amino acid, but this does not give any special radiolytic stability to the amino acid and the free radical localization [as a first reaction acts similar to Equation (4)] should occur in correspondence with the β-carbon atom as a result of the hydrogen abstraction so that the scheme of reaction Equation (4) can be applied, also in this case leading to the release of ammonia as the main reaction. Indeed, all the β-amino acids examined do not display any special radiolytic resistance in comparison to the other amino acids. The only exception is offered by dl-3-AIB, which can be separated from the others and whose Nγ% value is very close to that of α-AIB. The explanation of this special behavior of dl-3-AIB can be found in its chemical structure. The amino group is connected to a primary β-carbon atom rather than to the secondary carbon atom connection for all the other β-amino acids examined. Furthermore dl-3-AIB shows a methyl group connected to the α-carbon atom. This implies that the first reaction acting on dl-3-AIB is the H atom abstraction from the α-carbon atom and the free radical localization will occur at this site, which is a secondary carbon atom site rather than to the primary carbon atom site of the β-carbon. This means that the reaction of Equation (4) is hindered and the degradation path of α-carbon atom should follow only the reaction of Equation (7) leading to an enhanced radiostability of dl-3-AIB as already discussed for α-AIB.

### 3.5. Measurement of the Optical Activity and Optical Rotatory Dispersion (ORD)

The amino acids display at least an asymmetric center in their molecule which is the source of the optical activity of this class of molecules so that each amino acid molecule occurs in general as a couple of enantiomers. As discussed in several occasions in previous works [30,32,33,34,35,36,37,38,39,40,41,42,43,44], the radiolysis of an amino acid enantiomer causes the complete radiolysis of a fraction of the irradiated substrate but also its radioracemization. In other words, when an amino acid crystal is invested by high energy γ radiation part of the molecules present in it are directly degradated into other products as discussed above but, as shown in Equation (4), the formation of a free radical site may occur just in correspondence of the asymmetric carbon atom. The fate of this free radical site is described in Equation (5) where a disproportionation reaction occurs with the formation of an imine derivative and the re-formation of the starting amino acid. However, in this passage from the reactants of Equation (4) to the products of Equation (5), an inversion of the asymmetry center with the consequent passage of the molecular configuration from the L to D and *vice versa* could occur. The macroscopical effect of such an inversion of the asymmetric carbon atom is the racemization since in the original L amino acid starts to accumulate a certain amount of the D enantiomer with the consequent reduction in the optical activity and in the ORD. Bonner [60] has studied in great detail the radiation-induced racemization (commonly known as radioracemization) of amino acids and certain peptides showing that the racemization fraction which accumulates into the residual amino acid is about 5% and in general is not very sensitive to the radiation dose (especially at high dose).

Measurement of the optical activity by polarimetry, using the D line of a sodium lamp at 589 nm or the measurement of the ORD in a wider range of wavelengths is not a measurement of pure radioracemization of an irradiated substrate in general and an irradiated amino acid in particular. It is instead a measurement of the sum of the radioracemization and the radiolysis of the amino acid. As shown by Bonner [60], the radioracemization of a chiral substrate at high radiation dose is, after all, a secondary phenomenon since the primary effect of high energy radiation is the radiolysis, the complete degradation of the amino acid into smaller and achiral molecular fragments. Consequently, the irradiation of any chiral amino acid, such as l-ABA or d-ABA, leads to the complete degradation of the amino acids into NH_3_, CH_4_, H_2_, CO_2_, carboxylic, and keto acids. This molecular degradation is macroscopically manifested as a reduction in the optical activity and in the ORD and is what we call a false or apparent radioracemization since it is instead due to the disappearance of the molecules bearing the chiral center. An additional effect of the l-ABA irradiation is its partial conversion to d-ABA through the inversion of the chiral center as described in the reaction of Equation (8); this corresponds to a true radioracemization. From the work of Bonner [60] we know that this is a secondary effect in comparison to the radiolysis but the accumulation of a small amount of d-ABA compensates partially the optical activity (more precisely the optical purity) of l-ABA and contributes again to the general reduction of the optical activity and of the ORD curve. 

**Figure 6:**
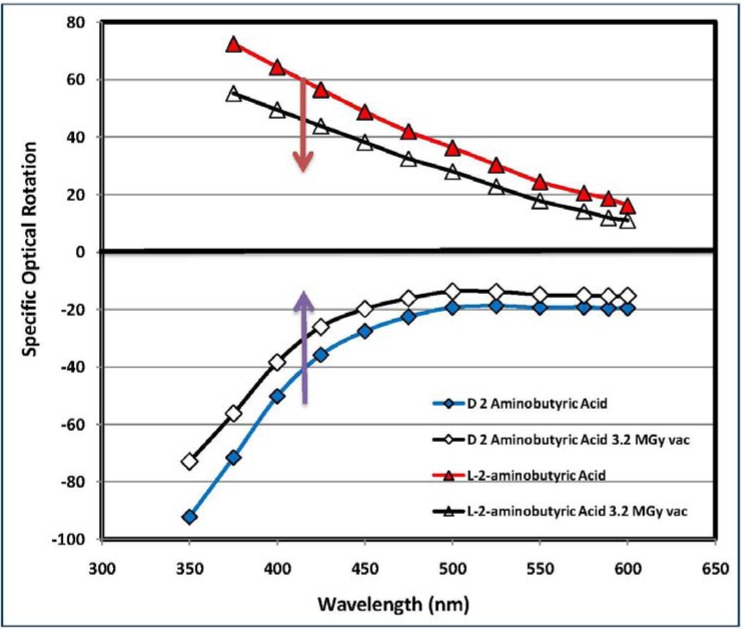
Optical Rotatory Dispersion (ORD) curves of l-ABA before radiolysis (red triangles) and after 3.2 MGy radiolysis (black dots). The enantiomer d-ABA in pristine state is represented by the blue diamonds connected with blue line, while the black line with the green squares is due to d-ABA after radiolysis at 3.2 MGy. Radiolysis and radioracemization causes the shift of the ORD curves toward the abscissa axis.

Figure 6 is a clear example of this phenomenon of combined apparent and true radioracemization of l-ABA and R-ABA: the radiolysis at 3.2 MGy causes a shift of the ORD curve toward the abscissa in the direction indicated by the arrows. This shift of the ORD curve toward abscissa axis is the result of the combined effect of radiolytic degradation of the amino acids and the partial racemization of part of the amino acids “survived” the radiolytic process. Using Equations (2) and (3) it is possible to evaluate the entity of the combined radiolytic degradation and racemization undergone by a given amino acid measuring the resulting amount of amino acid that “survived” the radiolysis and radioracemization process. The R_γ_% and R_α_% values at 3.2 MGy of all optically active amino acids studied are reported in Table 1. As discussed in previous works [30,32,33,34,35,36], there is in general a reasonable agreement between the Nγ% value measured by DSC and the R_γ_% and R_α_% values measured by ORD or polarimetry. Of course a complete agreement between the two measurements is not possible because Nγ% measures (through the DSC) only the residual amount of a given amino acid that “survived” the radiolysis process, irrespective for the enantiomeric excess. On the other hand, with R_γ_% and R_α_%, we estimate the amount of a given amino acid that “survived” the radiolysis process through the measurement of the residual enantiometic excess. It is evident that the values of R_γ_% and R_α_% are inaccurate and less reliable than Nγ%, since the ORD and polarimetry are not able to detect the racemized fraction of each amino acid that survived the radiolysis but which are not detectable with these optical methods. Furthermore, the radiolysis of amino acids could give as secondary products dimers and oligomers where the chiral center has been preserved. Such molecules may contribute to the R_γ_% and R_α_%, suggesting an unreliable higher purity of the radiolyzed amino acid in comparison to its Nγ% value.

On the other hand the importance of the ORD and polarimetric measurements reside in the fact that they give us an immediate sight of the degree of retention of the optical activity of chiral amino acids after a massive radiation dose of 3.2 MGy which corresponds to 23% of the total radiation dose the organic molecules will take from the beginning of the solar system formation until now (4.6 × 10^9^ years). The results shown in Table 1 are particularly encouraging in terms of preservation of the optical activity since all the amino acids examined in the present work show a very high level of residual optical activity notwithstanding the massive radiation dose administered.

## 4. Conclusions

A series of non-proteinogenic amino acids, most of them found in carbonaceous chondrites were irradiated in the solid state and in vacuum to a radiation dose of 3.2 MGy, equivalent to 23% of the total dose expected to be received by these molecules if embedded in asteroids, comets and other bodies of the solar system from its formation until now (4.6 × 10^9^ years).

The radiolyzed amino acids were studied by FT-IR spectroscopy in comparison to the pristine, non-irradiated amino acids. The FT-IR spectroscopy confirms the typical radiolytic degradation mechanism of aliphatic amino acids which is dominated by the release of ammonia and the formation of carboxylic and keto-acids as decomposition products.

The amount of each amino acid “survived” to the radiolytic process was measured by a DSC analytical technique using where necessary stainless steel-sealed crucibles rather than the conventional opened aluminum crucibles. All the amino acids examined show a good resistance to the radiolytic degradation and, among the amino acids studied, the best radiolytic stability is offered by α-AIB which is also the most common and most abundant amino acid found in carbonaceous chondrites. It is also found that the enantiomers l-ABA and d-ABA undergo the same level of radiolytic degradation as theoretically expected. Very small differences in the response to the radiolysis were attributed to the presence of impurities. β-Leucine together with norvaline and norleucine were proved to be the less resistant toward the action of radiation although 53.4%, 55.2% and 61.9% respectively were recovered unchanged after the radiolysis treatment at 3.2 MGy.

Polarimetric measurements and ORD measurements have confirmed that all the amino acids radiolyzed to 3.2 MGy under the form of a L or D enantiomer are able to preserve a high level of their original enantiomeric excess.

Based on all these results, it can be affirmed that it is not a surprise to find amino acids in carbonaceous chondrites and to find some of them in an enantiomeric excess even after 4.6 × 10^9^ years from their inclusion in asteroids and comets and even after 14 MGy of radiation dose released by radionuclide decay. In fact, an extrapolation of the results obtained at 3.2 MGy to 14 MGy shows that practically all amino acids can “survive” such a massive dose of radiation [35] in relatively small amounts.
